# Monosegmental fixation for the treatment of fractures of the thoracolumbar spine

**DOI:** 10.4103/0019-5413.36998

**Published:** 2007

**Authors:** Helton LA Defino, C Fernando PS Herrero, Carlos FEW Romeiro

**Affiliations:** Department of Biomechanics, Medicine and Rehabilitation of the Locomotor Apparatus, Faculty of Medicine of Ribeirão Preto, University of São Paulo, Brazil

**Keywords:** Monosegmental fixation, spinal fixation, spinal fractures, spinal trauma, thoracolumbar fractures

## Abstract

**Background:**

A short vertebral arthrodesis has been one of the objectives of the surgical treatment of fractures of the thoracolumbar spine. We present here clinical, functional and radiographic outcome obtained after monosegmental fixation (single posterior or combined anterior and posterior) of specific types of unstable thoracolumbar fractures.

**Materials and Methods:**

Twenty four patients with fractures of the thoracolumbar spine submitted to monosegmental surgical treatment (Group I - 18 single posterior monosegmental fixations and Group II - 6 combined anterior and posterior fixations) were retrospectively evaluated according to clinical, radiographic and functional parameters. The indication for surgery was instability or neurological deficit. All the procedures were indicated and performed by the senior surgeon (Helton LA Defino).

**Results:**

The patients from group I were followed-up from 2 to 12 years (mean: 6.65±2.96). The clinical, functional and radiographic results show that a single posterior monosegmental fixation is adequate and a satisfactory procedure to be used in specific types of thoracolumbar spine fractures, The patients from group II were followed-up from 9 to 15 years (mean: 13 ± 2,09 years). On group II the results of clinical evaluation showed moderate indices of residual pain and of satisfaction with the final result. The values obtained by functional evaluation showed that 66.6% of the patients were unable to return to their previous job and presented a moderate disability index (Oswestry = 16.6) and a significant reduction of quality of life based on the SF-36 questionnaire. Radiographic evaluation showed increased kyphosis of the fixed vertebral segment during the late postoperative period, accompanied by a reduction of the height of the intervertebral disk.

**Conclusion:**

It is possible to stabilize the fractures which have an anterior good load-bearing capacity by a standalone posterior monosegmental fixation. However this procedure, even with an anterior support is not suitable for fracture involving the vertebral body.

## INTRODUCTION

The execution of short vertebral arthrodesis has been one of the objectives of the surgical treatment of fractures of the thoracolumbar spine in order to preserve the intact vertebral segments that were not affected by traumatic injury. Monosegmental vertebral arthrodesis represents the maximum preservation of the vertebral segments during the execution of arthrodesis for the treatment of fractures and has been successfully performed for the treatment of specific types of fracture of the thoracolumbar spine.^1^–^4^

On the basis of biomechanical studies demonstrating that 80% to 90% of the axial compression forces in the erect position are absorbed by the anterior part of the spine (body and intervertebral disc), the recommended procedure is an anterior approach and reconstruction of the anterior portion of the vertebral segment whose weight-bearing capacity was damaged by the traumatic injury.^5^,^6^ In some specific types of fracture it is possible to maintain part of the fractured vertebral body, so that the reconstruction of the anterior vertebral segment will be limited only to one of the adjacent vertebral bodies.^2^,^3^,^4^,^7^

The objective of this retrospective study was to evaluate the clinical, functional and radiographic outcome obtained after monosegmental fixation (single posterior or combined anterior and posterior) of specific types of unstable thoracolumbar fractures.

## MATERIALS AND METHODS

Twenty four patients with unstable thoracolumbar fractures submitted to surgical treatment were retrospectively evaluated and divided into two groups: Group I (n=18)- patients were submitted to surgical treatment by single posterior monosegmental fixation and arthrodesis and Group II (n=6)- patients submitted to surgical treatment by combined posterior and anterior monosegmental fixation and arthrodesis. The instability was evaluated according to classical clinical, radiographic and other images examination. The indication of surgical treatment was directly related to the presence of instability of the injured vertebral segment and to neurological deficit. The decision to perform a posterior or a combined approach was related to the presence or absence or vertebral body fracture. The fractures with intact vertebral body were treated by posterior approach and fractures with vertebral body involvment by a anterior and posterior approach. No anterior or posterior decompression of the vertebral canal were performed even in patients with Frankel A grade because there was no bone compressing the spinal canal. Those patients had neurological déficit due to the dislocation of the vertebral segment.

**Figure 1:**
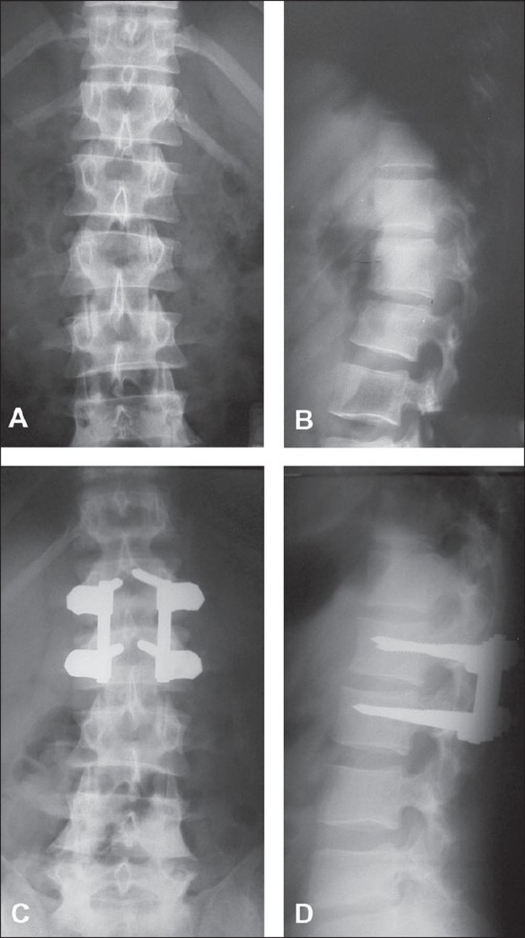
Preoperative X-rays of dorsolumbar spine AP and lateral (A & B) show a type B lumbar vertebral fracture. The patient was treated with a posterior monosegmental fixation- The immediate post operative X-ray AP and lateral (C & D) shows the posterior monosegmental fixation

**Figure 2:**
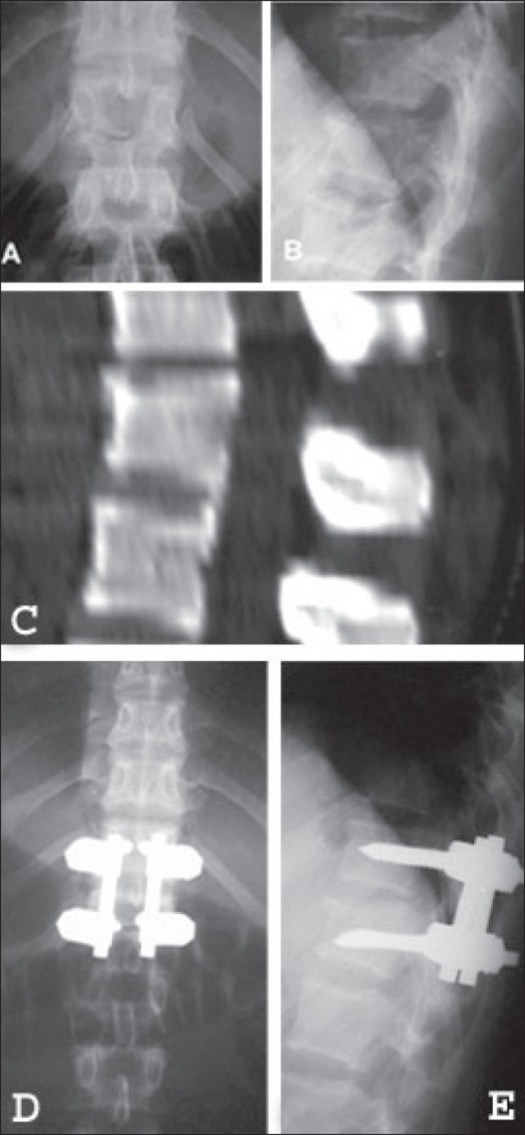
Preoperative X rays of dorsolumbar spine AP and lateral (A & B) shows a type B thoracolumbar fracture. Preoperative CT sagital reconstruction (C) shows the thoracolumbar fracture. The 2 years postoperative x-rays (D & E) show a posterior monosegmental fixation without complications

Group I [Table 1] patients were treated by single posterior monosegmental fixation and arthrodesis [Figures 1 and 2]. Seventeen patients were males and one was a female, ranging in age from 22 to 55 years (mean: 35.36 + 8.26). The causes of the fractures were: motor vehicle accidents (n=17, 94.4%) and fall from a height (n = 1, 5.5%). The lesion was localized in T10-T11 (n=2, 11,1%), in T11-T12 (n=4, 22,2%), in T12-L1 (n=6, 33,3%), in L1-L2 (n=2, 11,1%), in L2-L3 (n=1, 5,5%) and in L3-L4 (n=3, 16,6%). According to the classification of Magerl *et al.*,^8^ 11 fractures were of type B1 (61,1%), 3 of type B2 (16,6%), 1 of type B3 (5.5%) and 3 of type C (16.6%). The difference between this types is given in the Magerl's classification. The images examination allows to get information concerning the features of the fracture and allows its classification. In the specific case of type B3 fracture, the diagnosis was made using MRI.

**Table 1:** Shows patients in group I with level of the lesion, type of fracture according Magerl's classification; preoperative neurological status; level of fixation with, type of implant and followup

Patient	Lesion level	Type of lesion	Preoperative neurological deficit (Frankel)	Implant	Followup (years)
1	T10-T11	C1	A	USIS T10-11	8
2	T12-L1	B1	A	USIS T12-L1	7
3	T12-L1	B1	A	USIS T12-L1	9
4	T11-T12	B1	A	USS T11-12	6
5	T10-T11	B3	B	USS T10-11	5
6	T11-T12	B2	D	USS T11-12	2
7	T12-L1	B1	D	USS T12-L1	5
8	T12-L1	B1	E	USIS T12-L1	9
9	L3-L4	B2	E	USS L3-4	5
10	L1-L2	B1	E	IF L1-L2	4
11	L2-L3	B2	E	USIS L2-3	8
12	T11-T12	B1	E	USIS T11-T12	10
13	T12-L1	B1	E	USIS T12-L1	12
14	L1-L2	C2	E	IF L1-L2	4
15	L3-L4	C2	E	USS L3-4	3
16	T12-L1	B1	E	IF T12-L1	2
17	T11-T12	B1	E	USIS T12-11	8
18	L2-L3	B2	E	USIS L2-3	10

The classification of the fractures was performed by Radiograph and CT or MRI when required. The used classification pattern was the same for all patients and it was performed by the senior author. Evaluation of neurological status according to the classification of Frankel *et al.*,^9^ showed that 11 patients belonged to group E (61.1%), two to group D (11.1%), one to group B (5.5%) and four to group A (22.2%). Combined injuries such as long bone fracture and abdominal trauma were present in six patients (33.3%).

Single posterior monosegmental fixation was indicated in type B and C fractures according to the classification of Magerl *et al.*,^8^ with an intact vertebral body for load support. The standalone posterior fixation was performed only in patients with B1 (n=11) or B3 (n=1) fractures. It means that the vertebral body was intact. All patients were treated by monosegmental posterior fixation and arthrodesis of the injured vertebral segment using an autogenous cortico-cancellous bone graft removed from the posterior iliac bone. The vertebral segment was stabilized by bilateral pedicular fixation using the USIS (Ulrich) system of vertebral fixation in nine patients (50%), 6.0 mm USS pedicular screws (Synthes) in six (33.3%) and the Internal Fixator (Synthes) in three (16.6%) [Table 1]. This being a retrospective study, the use of the implants reflects the evolution of the spinal implants in the last decade.

**Figure 3:**
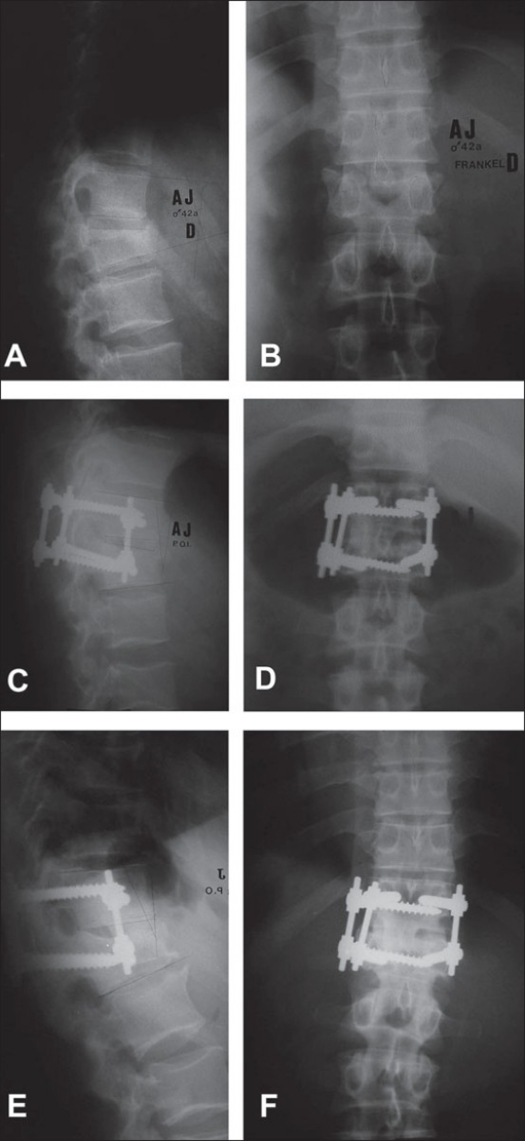
Preoperative X rays of dorsolumbar spine AP and lateral (A & B) show a lumbar fracture of type A. Immediate postoperative X ray (C & D) and after 13 years radiographic follow-up (E & F) show fracture consolidation after monosegmental fixation with a combined approach

**Figure 4:**
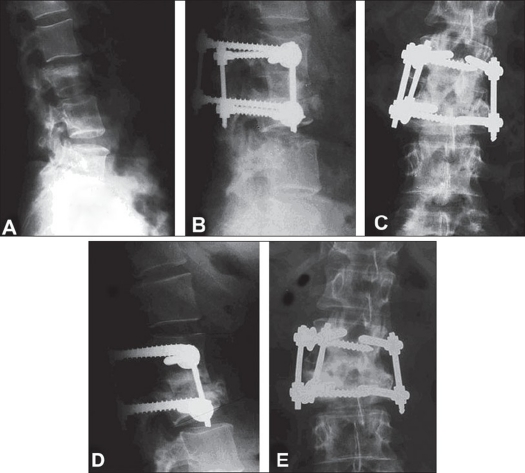
Preoperative X ray of dorsolumbar spine (A) shows a vertebral fracture of type A. The patient was treated with a monosegmental fixation through a combined approach. Immediate postoperative X rays AP and lateral (B & C) show the combined monosegmental fixation and the X rays after nine years follow-up (D & E) show the fracture consolidation

Group II [Table 2] Consists of six patients who had anterior vertebral body involvement and were treated by combined anterior and posterior monosegmental fixation and arthrodesis [Figures 3 and 4]. Patient age ranged from 32 a 45 years (mean: 37,6 years); four patients were males and two females. The causes of the fractures were: motor vehicles accidents (n=4) (66.6%) and fall from a height (n=2) (33.3%). The fracture was located at the T12 level (n=1) (16.6%), L1 level (n=2) (33.3%), L2 level (n=2) (33.3%) and at the L3 level (n=1) (16.6%). According to the classification of Magerl *et al.*,^8^,^10^ two fractures were type B1 (33.3%), one was type B2 (16.6%), two were type C1 (33.3%) and 1 type C2 (16,63%). Evaluation of neurological signs and symptoms according to the classification of Frankel *et al.*,^11^ showed that three patients belonged to group E (50%), two to group D (33.3%) and one to group A (16.6%). Associated injuries such as long bone fractures and abdominal trauma were present (n=2) (33,36%) [Table 2].

**Table 2:** Shows details of patients of Group II - Age, sex, mechanism, level of the lesion, type of fracture according Magerl's classification, preoperative neurological status level of fixation and type of implant and patients followup

Patient	Age	Sex	Mechanism	Lesion level	Type of lesion	Neurological deficit (Frankel)	Implant	Followup (years)
1	45	M	Fall	L2	C1	A	USIS L2-L1	13
2	40	M	Fall	L2	C2	D	USIS L2-L3	14
3	30	F	Car	L1	B2	D	USIS L1-T12	15
4	36	F	Car	L3	C1	E	USIS L3-L2	9
5	32	M	Car	L1	B2	E	USIS L1-T12	13
6	43	M	Car	T12	B2	E	USIS T12-T11	14

All patients were treated by combined monosegmental fixation of the injured vertebral segment associated with posterior and anterior arthrodesis using an autologous cortico-cancellous bone graft obtained from the iliac crest. The vertebral segment was stabilized by bilateral posterior pedicular fixation and by anterior fixation using the USIS (Ulrich) system of vertebral fixation in all six patients. Anterior arthrodesis was performed by removing the intervertebral disc and part of the fractured vertebral body and by placement of a cortico-cancellous bone graft for the restoration of the anterior part of the vertebral segment. The anterior approach was performed by an open procedure. The surgical approach depended on the level of the fracture. The decompression of the canal was (either anterior or posterior) not performed as there was no bony compression in the spinal canal.

During the postoperative period, the patients did not use external immobilization and there was no limitation of walking or rehabilitation in both the groups. The patients from both the groups were evaluated on the basis of clinical and radiological parameters and by functional evaluation. The parameters used in clinical evaluation were neurological according to the scale of Frankel *et al.*,^11^ subjective results by the patients themselves (good, fair, poor) and evaluation according to the Denis pain scale^12^ [Table 3].

**Table 3:** Denis pain scale

P1: No pain
P2: Occasinal minimal pain; no need for medication
P3: Moderate pain, occasionally medications e no interruption of work or activities of daily living
P4: Moderate to severe pain, occasionally absent from work, significant changes in activities of daily living
P5: Constant, severe pain; chronic pain medications

The parameters used in the radiographic study were: measurement of initial kyphosis of the injured vertebral segment, correction obtained during the immediate postoperative period and its maintenance during late followup. The consolidation of arthrodesis, the bone reabsorption around the implants and the possible changes in the fixation material were also analyzed. The consolidation of arthrodesis was evaluated logically. The consolidation of the graft and the appearance of a graft bone bridge between the vertebral segment was considered a good result. Functional evaluation was performed using the Denis work scale^12^ [Table 4], the Oswestry disability index^9^ and the general health evaluation questionnaire SF-36.^13^

**Table 4:** Denis work scale

W1: Return to previous employment (heavy labor) or physically demanding activities
W2: Able to return to previous employment (sedentary) or return to heavy labor with restrictions
W3: Unable to return to previous employment but works full time at new job
W4: Unable to return to full time work
W5: No work, completely disabled

Data regarding the measurements of the kyphosis angle of the injured vertebral segment during the preoperative period and the initial and late postoperative period were analyzed statistically on group I by analysis of variance (ANOVA) for repeated measures followed by the Bonferroni post hoc test, with the level of significance set at p ≤ 0.05 and on group II by Student t-test after verification of sample normality by the Kolmogorov-Smirnov test.

## RESULTS

**Figure 5:**
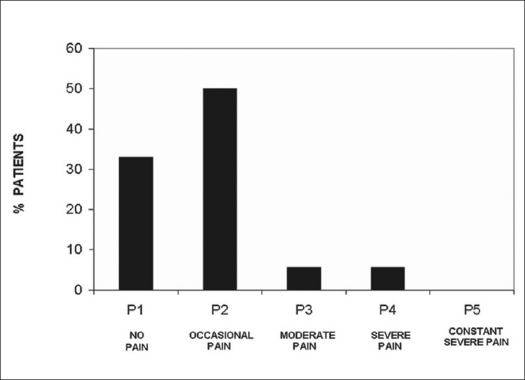
Bar-diagram shows long-term follow-up results according to Denis pain scale on group I

In group I the followup ranged from two to 12 years (mean: 6.65 ± 2.93). Upon clinical evaluation, one patient progressed from Frankel D to E and the remaining ones did not show any neurological changes. In the subjective evaluation performed by the patients, the final result was considered to be good by 14 of them (77.7%), fair by 3 (16.6%) and poor by 1 (5.5%). Pain evaluation according to the Denis scale^10^ showed that six patients had no pain (33.3%), nine had occasional pain (50%), one had moderate pain (5.5%) and one had moderate to intense pain (5.5%) [Figure 5].

Radiographic evaluation revealed that kyphosis of the fractured vertebral segment ranged from 0° to 36° (mean: 14.4° ± 9.62) during the preoperative period, from 10° of lordosis to 20° of kyphosis (mean: 3.72 ± 6.73° of kyphosis) during the immediate postoperative period and from 10° of lordosis to 20° of kyphosis during the late postoperative period (mean: 7.72 ± 7.73° of kyphosis).

On late evaluation all patients, except one, presented a reduction of at least 50% in the height of the intervertebral disk of the fixed vertebral segment. In four patients (22.2%), reabsorption was observed around the implant, but with no signs of break of the bit latter. The incorporation of the bone graft and arthrodesis consolidation was observed in all patients, with no case of pseudoarthrosis [Table 5].

**Table 5:** Longterm radiological evaluation of the patients of the group I showing the segmental kyphosis at the preoperative, postoperative and longterm followup; the reduction of disc height at the vertebral segment fixated; the bone reabsorption around the screws and the consolidation of the arthrodesis

Patient	Kyphosis			Disc height reduction (%)	Bone reabsorption	Arthrodesis
				
	Preoperative	Immediate-postoperative	Long term followup
1	10	6	18	> 50	No	Yes
2	19	8	12	50	No	Yes
3	3	5	2	> 50	Yes	Yes
4	15	0	4	> 50	No	Yes
5	10	−4	0	> 50	Yes	Yes
6	22	10	16	> 50	No	Yes
7	30	8	11	> 50	Yes	Yes
8	6	3	11	50	No	Yes
9	36	11	15	> 50	No	Yes
10	10	3	4	> 50	No	Yes
11	19	2	11	> 50	No	Yes
12	12	−6	−3	> 50	No	Yes
13	0	−10	−10	50	No	Yes
14	5	1	3	Zero	Yes	Yes
15	25	20	20	50	No	Yes
16	10	4	5	50	No	Yes
17	20	4	8	50	No	Yes
18	8	2	4	50	No	Yes

A statistically significant difference was observed between the initial kyphosis of the injured vertebral segment and the kyphosis observed in the immediate postoperative period (*P* < 0.001). A significant difference was also observed between the kyphosis values of the injured vertebral segment measured during the immediate postoperative period and those measured during the late period (*P* = 0.001) and between those measured during the preoperative period and the late postoperative period (*P* = 0.001). The postoperative data were acquired at the time of discharge of patients. The late period is the last examination of the patient and is coincident with the individual patient followup.

**Figure 6:**
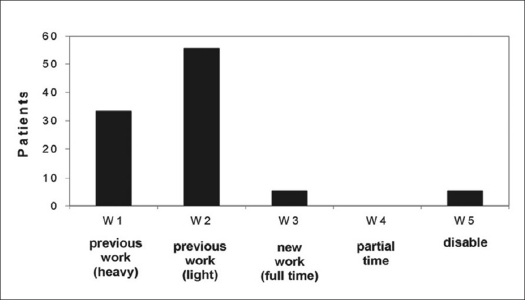
Bar-diagram shows long-term results according to Denis work scale on group I

**Figure 7:**
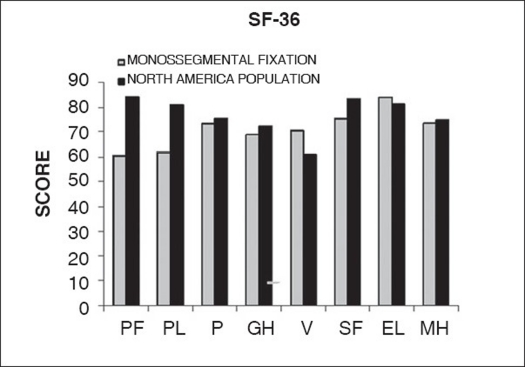
Bar-diagram shows long-term results of monosegmental fixation compared to General Health Survey SF-36 average score of North America Population on group I. PF: Phisical function; PL: Phisical limitation; P: pain; GH: general health: V: vitality; SF: Social function; EL: Emotional limitation; MH: Mental health

Functional evaluation showed that six patients returned to their previous job involving intense physical activity (33.3%), 10 returned to their previous job involving light physical activity (55.5%), one was forced to change his job but continued to work on a full time basis (5.5%) and one was unable to return to work (5.5%) [Figure 6]. The mean Oswestry disability index^2^,^14^ obtained was 10.33 ± 10.87 (range: zero to 42) [Figure 7] The mean scores obtained with the SF-36 general health survey questionnaire,^15^,^16^ for which the best possible score is 100, were: 60.29 ± 28.33 for physical function, 61.66 ± 38.34 for functional limitation, 73.88 ± 24.87 for pain, 68.61 ± 13.91 for general health, 70.5 ± 10.69 for vitality, 75 ± 18.68 for social function, 84.21 ± 24.61 for emotional limitation and 73.77 ± 8.81 for mental health [Table 6].

**Table 6:** Results of Oswestry disability index (Roland. M. and Fairbank. J, 2000) compared to longterm results of monosegmental fixation on group I

1-	“Normal” polpulation	10,19%
2-	Pelvic fractures	13,26%
3-	Spondilolistesis	26,63%
4-	Cronic lumbar pain	43,3%
5-	Monosegmental fixation	10,33%

**Figure 8:**
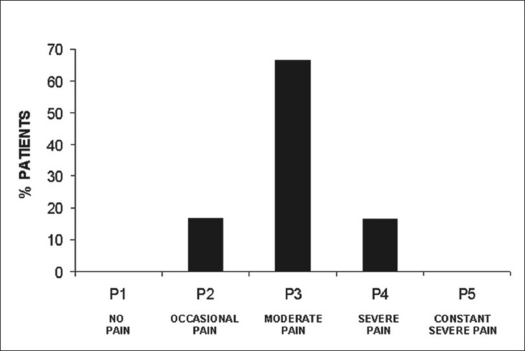
Bar-diagram shows long-term follow-up results according Denis pain scale on group II

In group II, patient followup ranged from 9 to 15 years (mean: 13 ± 2,09 years). Clinical evaluation did not reveal changes in the initial neurological picture. In the subjective evaluation three patients (50%) considered the final result of treatment to be good, one (16.6%) considered it fair and two (33.3%) considered it poor. In the clinical evaluation of pain by the Denis scale,^12^ one patient presented occasional pain (16.6%), four patients presented moderate pain (66.6%) and one patient presented moderate to intense pain (16.6%) [Figure 8].

**Figure 9:**
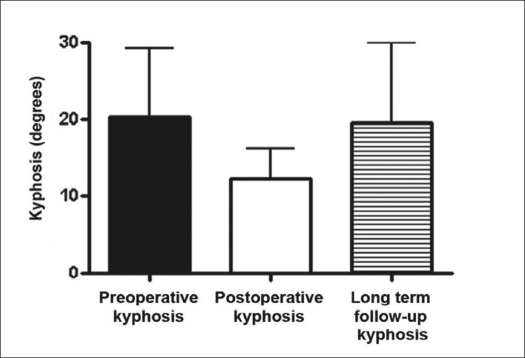
Bar-diagram shows radiographic kyphosis evaluation of the injured segment from the patients of the group II

Kyphosis of the fractured vertebral segment ranged from 7° to 30° (mean: 20.33 ± 90) during the preoperative period, from 7° to 20° (mean: 12.3 ± 4.0) during the immediate postoperative period and from 7° to 38° (mean: 19.5 ± 10.5) during late followup [Figure 9]. No significant difference was observed between the preoperative, immediate postoperative and late postoperative values of kyphosis of the fractured vertebral segment. However, it should be pointed out that the small sample size may have interfered with the statistical evaluation.

Reabsorption around the implants was not observed in any patient, nor were any signs of breaking of the material. The incorporation of the bone graft was observed in all patients, with no case of pseudoarthrosis.

**Figure 10:**
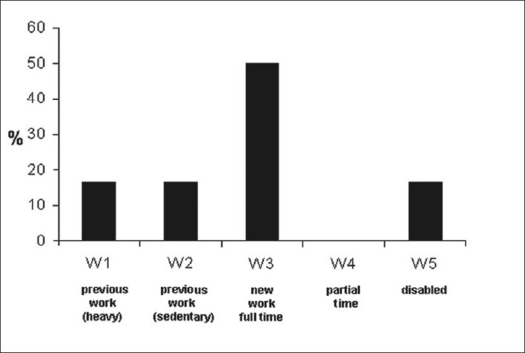
Bar-diagram shows long-term results according Denis work scale on group II

Functional evaluation according to the Denis scale showed that one patient returned to his previous job involving intense physical activity (16.6%), one patient returned to his previous job involving light physical activity (16.6%), three patients had to change their job but were able to work full-time (50%) and one patient was unable to return to work (16.6%) [Figure 10].

**Figure 11:**
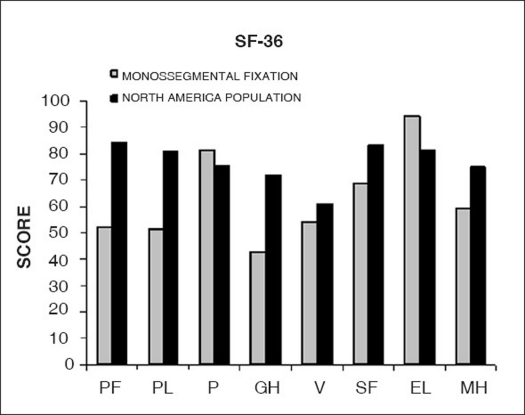
Bar-diagram shows long-term results of monosegmental fixation compared to General Health Survey SF-36 average score of North America Population on group II PF: Phisical function; PL: Phisical limitation; P: pain; GH: general health: V: vitality; SF: Social function; EL: Emotional limitation; MH: Mental health

The mean Oswestry disability index^9^ obtained was 16.6% [Table 7] and the mean scores for the general health SF-36 questionnaire^13^ were: 52.2 for physical function, 51.4 for functional limitation, 81.4 for pain, 42.7 for general health, 54.2 for vitality, 68.7 for social function, 94.3 for emotional limitation and 59.5 for mental health, with 100 being the highest possible score [Figure 11].

**Table 7:** Results of Oswestry disability index (Roland. M. and Fairbank. J, 2000) compared to longterm results of monosegmental fixation on group II

1-	“Normal” polpulation	10,19%
2-	Pelvic fractures	13,26%
3-	Spondilolistesis	26,63%
4-	Chronic lumbar pain	43,3%
5-	Monosegmental fixation	16,66%

## DISCUSSION

Surgical treatment of the thoracolumbar spine fractures has been classically based on arthrodesis involving the fractured vertebral segment and some intact adjacent vertebral segments. Better understanding of the biomechanics of the vertebral segment, more detailed and precise classification of the injuries and the development of fixation systems that provide better mechanical stability have permitted to reach the current objectives of such treatment, among them short arthrodesis for the preservation of the non-injured segments of the spine and elimination of the use of external immobilization.^1^–^4^,^14^,^17^

Monosegmental arthrodesis represents the maximum preservation of the intact vertebral segments adjacent to the fractured vertebra and its execution has become possible with the development of systems of pedicular fixation. The use of fixation and single posterior monosegmental arthrodesis for the treatment of thoracolumbar spine fractures is the result of the practical application of current knowledge in the field of spinal surgery. The biomechanical concepts support the execution of monosegmental fixation for the restoration of the damaged posterior tension band as long as there is integrity of the vertebral body for weight support.^5^,^6^

Despite the controversy existing about the treatment of the thoracolumbar spine fractures, the group I fractures may be one of the few about which there is not much discussion regarding the indication of surgical treatment. These patients in our series presented with type B or C fractures as per the classification of Magerl *et al.*, which involves associated injury of the posterior vertebral ligaments, bony structures and paraspinous muscles. The concept of the inability of these injuries to heal and of the vertebral segment stability to be reestablished has been well defined.^4^ The injuries are unstable, progress with post-traumatic deformity when treated conservatively and there is no controversy about the indication of surgical treatment in these types of injuries.

In group II, loss of height of the space between the injured vertebral bodies submitted to arthrodesis was observed in all patients and was filled by the bone graft. The reduced space between the fused vertebral bodies was probably due to sinking of the bone graft into the fractured vertebral body, whose intact portion not affected by the injury did not present the mechanical properties necessary for anterior weight-bearing. Cage subsidence has been observed in non-fractured vertebral bodies after the removal of the vertebral end plate and both features can be explained by the lack of weight-bearing ability of cancellous bone of the vertebral body despite the maintenance of its integrity.

The current view about the importance of the maintenance of the vertebral end plate for weight bearing permits us to understand the cause of the loss of height of the segmental arthrodesis performed.^15^ However, Been *et al.*,^7^ observed a satisfactory clinical and radiographic outcome with surgical treatment of post-traumatic thoracolumbar kyphosis after simple type A fractures employing a monosegmental anterior procedure alone or a combined anterior and posterior procedure. They did not observe sinking of the graft into the vertebral body and kyphosis of the fractured vertebral segment was maintained. This observation must be related to the consolidation and reorganization of the compacted bone of the fractured vertebral body after the development of post-traumatic deformity. This situation differs from that observed in our patients, who were operated during the acute phase, explaining the difference in the results obtained with the same type of treatment. Schultheiss *et al.*,^18^ also reported poor results of monosegmental arthrodesis for the treatment of this type of fracture. However, they used solvent-preserved bovine cancellous bone blocks and attributed the poor results to the type of graft rather than to the type of procedure. They reported integration of the graft and absence of the loss of correction in eight out of 11 patients who had received an autogenous iliac crest graft.

In the radiological evaluation of our patients correlated with functional evaluation, poor clinical and functional results were observed together with loss of height of the vertebral segment submitted to arthrodesis. However, a lack of correlation between clinical and functional results has been frequently reported for spinal fractures.^14^,^17^ Although, even in the presence of good clinical and functional results, a combined approach to the injured vertebral segment does not appear to be justified in order to obtain radiological results that are unsatisfactory.

On the other hand, single posterior monosegmental arthrodesis for patients with unstable lesions of type B or C according to the classification of Magerl^8^,^10^ and without loss of the weight-bearing capability of the vertebral body has yielded good clinical and functional results and continues to be our preferred method for the surgical treatment. Increased kyphosis of the fractured vertebral segment was also observed in the patients of group I and was directly related to reduction of the height of the injured intervertebral disc.

## CONCLUSIONS

Single posterior monosegmental arthrodesis is a procedure that can be used for the treatment of complex injuries of the vertebral segment (luxations) as long as the vertebral body does not present a fracture–, which substantially impairs weight-bearing capability of the anterior column, as seen in burst fractures like e.g., type A3.3.^8^

Combined anterior and posterior fixation and arthrodesis of unstable thoracolumbar fractures with involvement of the vertebral body led to radiographically observed loss of correction during the postoperative followup.
